# Test your knowledge and understanding

**Published:** 2015

This page is designed to help you test your own understanding of the concepts covered in this issue, and to reflect on what you have learnt. We hope that you will also discuss the questions with your colleagues and other members of the eye care team, perhaps in a journal club. To complete the activities online – and get instant feedback – please visit **www.cehjournal.org**

**Table:** 

**1.**	**The BETTS classification has been introduced to standardise classification of ocular injuries. This simplified system can NOT be used to:**	**Select one**
**a**	Audit ocular injuries at a hospital	□
**b**	Assist with visual prognosis in conjunction with the ocular trauma score	□
**c**	Assess trauma with intraocular foreign body	□
**d**	Describe chemical injuries	□
**2.**	**In assessing a patient with ocular trauma, the patient is most likely to be in a state of anxiety. What is the most appropriate action to take to manage the anxiety?**	**Select one**
**a**	Adopt a calm, sympathetic, reassuring and yet authoritative presence	□
**b**	Take a quick visual acuity and make a prognosis	□
**c**	Establish with some urgency who and what caused the trauma	□
**d**	Provide intravenous analgesics/painkillers immediately	□

## ANSWERS

**1. D.** BETTS is tailored to describe mechanical injuries to the eye globe. All definitions are in relation to corneal and scleral tissue penetration. It does not assist with classifying chemical injuries.
**2. A.** It is important to manage oneself first when trying to deal with another person's anxiety. Even if the eye injury is very bad, avoid giving that indication at the beginning, whether by what you say to the patient or to your colleagues. It is often necessary to stabilise the patient prior to any examination or the use of painkillers.

